# Ileal Interposition in Rats with Experimental Type 2 Like Diabetes Improves Glycemic Control Independently of Glucose Absorption

**DOI:** 10.1155/2015/490365

**Published:** 2015-06-22

**Authors:** Christian Ferdinand Jurowich, Christoph Otto, Prashanth Reddy Rikkala, Nicole Wagner, Ivana Vrhovac, Ivan Sabolić, Christoph-Thomas Germer, Hermann Koepsell

**Affiliations:** ^1^Department of General, Visceral, Vascular and Paediatric Surgery, University Hospital of Würzburg, Oberdürrbacher Straße 6, 97080 Würzburg, Germany; ^2^Institute of Anatomy and Cell Biology, University of Würzburg, Koellikerstraße 6, 97070 Würzburg, Germany; ^3^Molecular Toxicology Unit, Institute for Medical Research & Occupational Health, Ksaverska Cesta 2, 10000 Zagreb, Croatia; ^4^Department of Molecular Plant Physiology and Biophysics, Julius-von-Sachs-Institute, University of Würzburg, Julius-von-Sachs-Platz 2, 97082 Würzburg, Germany

## Abstract

Bariatric operations in obese patients with type 2 diabetes often improve diabetes before weight loss is observed. In patients mainly Roux-en-Y-gastric bypass with partial stomach resection is performed. Duodenojejunal bypass (DJB) and ileal interposition (IIP) are employed in animal experiments. Due to increased glucose exposition of L-cells located in distal ileum, all bariatric surgery procedures lead to higher secretion of antidiabetic glucagon like peptide-1 (GLP-1) after glucose gavage. After DJB also downregulation of Na^+^-d-glucose cotransporter SGLT1 was observed. This suggested a direct contribution of decreased glucose absorption to the antidiabetic effect of bariatric surgery. To investigate whether glucose absorption is also decreased after IIP, we induced diabetes with decreased glucose tolerance and insulin sensitivity in male rats and investigated effects of IIP on diabetes and SGLT1. After IIP, we observed weight-independent improvement of glucose tolerance, increased insulin sensitivity, and increased plasma GLP-1 after glucose gavage. The interposed ileum was increased in diameter and showed increased length of villi, hyperplasia of the epithelial layer, and increased number of L-cells. The amount of SGLT1-mediated glucose uptake in interposed ileum was increased 2-fold reaching the same level as in jejunum. Thus, improvement of glycemic control by bariatric surgery does not require decreased glucose absorption.

## 1. Introduction

The global prevalence of type 2 diabetes which is often associated with obesity is increasing dramatically [1]. Bariatric surgery proved to be the most effective long-term treatment for obesity [2–6] and has been shown to improve glucose homeostasis independently of weight loss [7, 8]. The International Diabetes Federation recommends bariatric surgery for treatment of obese patients with a BMI >35 and of patients with poorly controlled diabetes and a BMI between 30 and 35 [9]. Bariatric procedures performed in patients and tested in animals include bypass of the foregut (duodenal-jejunal bypass (DJB)) [10, 11], bypass of the foregut in combination with partial or total removal of the stomach (Roux-en-Y-gastric biliary bypass (RYGB)) [2], and interposition of a segment of the distal ileum into the proximal jejunum (ileal interposition (IIP)) [12, 13]. These surgery procedures improve glycemic control and decrease body weight. Considering that there are various routes and mechanisms of the gut involved in regulation of energy balance and metabolism [14, 15], the different surgical procedures are expected to improve glycemic control and decrease body weight in somewhat different ways.

Nutrient-dependent regulation of metabolism by the gut includes effects on food uptake, nutrient absorption, energy balance, metabolism, and glycemic control. It involves effects of enterohormones and neuronal signals from the gut on central regulations of food intake and energy balance, effects of enteric nerves on motility and secretion of digestive enzymes, effects of enterohormones on secretion of insulin and amylin, effects on metabolism, and nutrient-dependent regulation of small intestinal transporters [6, 14–25]. Previous investigations found that DJB reduces food uptake and increases insulin sensitivity and secretion of glucagon like peptide-1 (GLP-1) and peptide YY (PYY) [26–29]; RYGB reduces food intake and increases energy expenditure, insulin sensitivity, and secretion of GLP-1, PYY, glucagon like peptide-2 (GLP-2), and ghrelin [30–36]; and IIP improves insulin sensitivity, increases release of GLP-1 and PYY, and accelerates small intestinal bile acid absorption [12, 26, 27, 29, 30, 37–48].

All of the aforementioned bariatric procedures are supposed to cause postprandial elevation of L-cell secretagogues, including glucose, peptides, and short chain fatty acids in ileum and colon where the L-cells are located. L-cells express proglucagon-derived peptides and PYY. Stimulation of postprandial secretion of GLP-1 and PYY appears to be common to the described bariatric procedures. Via interaction with the GLP-1 receptor, GLP-1 stimulates glucose-dependent insulin secretion by pancreatic *β* cells, stimulates insulin biosynthesis, has a trophic effect on *β* cells, inhibits glucagon secretion in *β* cells, and reduces appetite in the central nervous system [21]. In addition presumed indirect effects of GLP-1 such as improvement of insulin sensitivity, reduction of hepatic gluconeogenesis, and increase of glucose use in skeletal muscles have been described [20, 49]. Because the bariatric procedures change the postprandial time concentration profiles of nutrients in small intestine, they are also thought to change the nutrient-dependent regulations of small intestinal regeneration, protein expression, and metabolism [32, 50–52]. Bariatric surgery procedures like RYGB that include partial or total removal of the stomach alter regulatory mechanisms governed by the stomach such as increase of appetite by secretion of ghrelin and regulation of secretion of bile acid and pancreatic enzymes. These procedures are supposed to impair the vagal nerve integrity more strongly compared to DJB and IIP, which may impair the gut-brain cross talk [14, 15]. At variance with RYGB and DJB, IIP is not thought to dramatically change food passage velocity. Like DJB, IIP is not thought to change regulatory mechanisms governed by the stomach. Thus, IIP may be a good model for elucidating gut-related mechanisms regulating energy balance and metabolism to improve diabetic control by bariatric surgery.

Because stimulation of GLP-1 secretion by bariatric surgery in animal models has been mostly demonstrated in response to gavage with glucose [27, 28, 40, 42, 44, 47, 48], physiological relevance of glucose-dependent stimulation of GLP-1 secretion is implicated. This view is supported by recent data showing that pharmacological inhibition of Na^+^-d-glucose cotransporter SGLT1 in small intestine, which is rate limiting for intestinal glucose absorption [25], leads to increased glucose-dependent secretion of GLP-1 and improves glycemic control in type 2 diabetes [53, 54]. Recently, we reported that DJB in diabetic rats was followed by downregulated expression of the Na^+^-d-glucose cotransporter SGLT1 in jejunum [11]. Thus, the glucose concentration in the distal ileum, where most L-cells are localized, is increased not only due to missing glucose absorption in the bypassed proximal jejunum but also due to downregulation of SGLT1. We have discussed the possibility that the antidiabetic effect of DJB and other bariatric surgery procedures may not only be due to increased glucose-induced GLP-1 secretion but also result from a slowed down and reduced increase of blood glucose after glucose ingestion. This may influence glucose metabolism in the liver that is changed in type 2 diabetes mellitus [11, 55–57]. To test this hypothesis, we investigate in the present study whether SGLT1-mediated glucose transport in small intestine of diabetic rats which is rate limiting for intestinal glucose absorption is also downregulated after IIP and may contribute to the antidiabetic effect in addition to improved exposure of L-cells to glucose. Because morphological changes of the interposed ileum after IIP have been described but not investigated in detail [12, 58] and changes in glucose metabolism associated with morphological changes after RYGB have been described [52], we also investigated the morphology of the interposed ileum by light and electron microscopy and determined the expression of SGLT1 protein in the enterocytes.

## 2. Materials and Methods

### 2.1. Animals

Male Lewis rats aged 7 weeks with 180–200 g body weight were obtained from Harlan Laboratories GmbH (Venray, Netherlands). The animals were maintained in groups of 2–4 animals in a pathogen-free environment under constant ambient temperature and humidity in a 12-hour light-dark cycle with free access to food and water unless otherwise stated. The study was approved by the Animal Care Committee of the local government in accordance with national guidelines for animal care (German Law for the Protection of Animals).

### 2.2. Diets

The hypercaloric high-fat diet (HFD, altromin number 40003) contained 18.2% protein, 22.1% fat, 33.5% polysaccharides, 9.8% disaccharides, and 1.7% monosaccharides. The values represent percentage of wet weight.

### 2.3. Streptozotocin- (STZ-) Induced Diabetes

In male rats kept for three weeks on HFD (*n* = 18) diabetes was induced by an intraperitoneal injection of 35 mg/kg STZ as described [11, 59, 60].

### 2.4. Interventions

**Figure 1:**
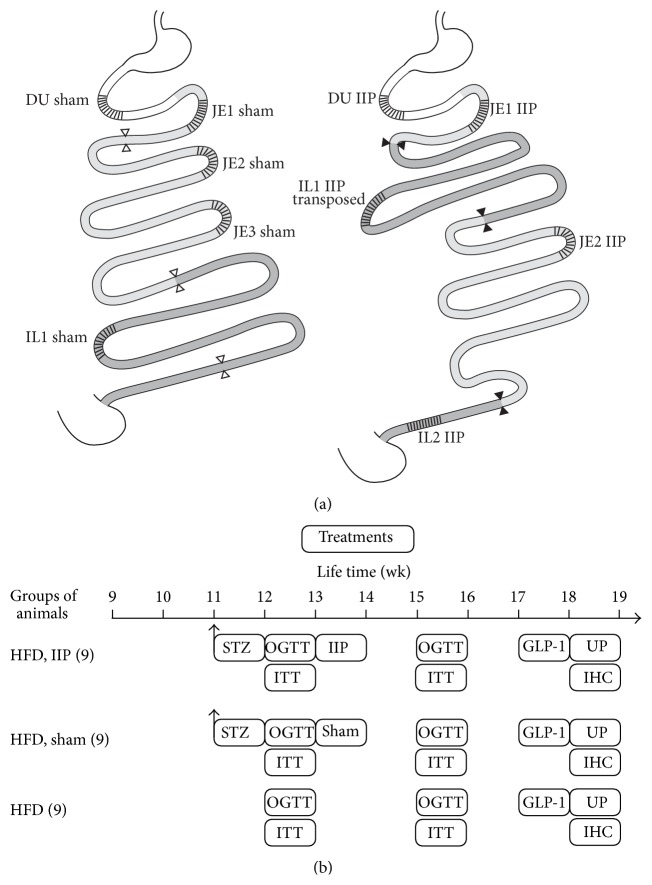
Schematic drawing of the performed ileal interposition (IIP) surgery (a) and the experimental protocols (b). (a) Stomach and duodenum (DU) are indicated in white, jejunum (JE) is in light grey, and ileum (IL) is in dark grey. The unchanged anatomy after sham operation is shown on the left and the anatomy after IIP on the right. Regions that were analyzed for glucose uptake are cross-hatched. (b) The performed experimental procedures and measurements. Beginning with the 9th week of life, all animals were kept on high-fat diet (HFD). The number of employed animals is indicated in parenthesis. STZ, streptozotocin injection; GLP-1, measurements of GLP-1; UP, uptake measurements; IHC, immunohistochemistry and electron microscopy.

Surgery was performed in 18 male rats after an overnight fast. Anesthesia was induced and maintained with isoflurane as described previously [11]. After midline laparotomy, IIP surgery was performed in 9 animals by isolating a 10 cm ileal segment 5 cm oral to Bauhin's valve. The remaining ileal ends were reconstructed by termino-terminal ileoileostomy in single-stitch-technique. The isolated ileal segment was interposed to the proximal jejunum 5 cm aboral to the duodenojejunal flexure. The jejunum was transected. Jejunoileostomy and the ileojejunostomy were performed in single-stitch-technique (termino-terminal, isoperistaltic) (Figure 1(a)). Sham operation was performed in 9 animals. It included median laparotomy, transverse transection of the ileum, and sewing by single-stitch-technique.

### 2.5. Study Protocol

An overview of the study protocol is shown in Figure 1(b). Male Lewis rats (*n* = 27) received HFD between the 9th and the 18th weeks of life. At 11 weeks of life, 18 animals were treated with STZ. At 12 weeks of life, an oral glucose tolerance test (OGTT) and an insulin tolerance test (ITT) were performed in all animals. At 12 weeks of life, ileal interposition (IIP) or sham operation was performed on STZ-treated animals (*n* = 9, each). At 15 weeks of life, a second OGTT and ITT were performed in all animals. At 17 weeks of life, glucose-induced secretion of GLP-1 was measured. One week later, the animals were sacrificed and the different segments of small intestine were used for* ex vivo* measurements of glucose uptake, immunohistochemistry, and electron microscopy.

### 2.6. Antibodies

Polyclonal antibodies against amino acids 585–600 (PKDTIEIDAEAPQKEK) of rat SGLT1 (rSGLT1-Ab) were raised in rabbits and affinity-purified using the respective antigenic peptide as described [61]. Polyclonal antibody against glucagon like peptide-1 (C-17, sc-7782, and GLP-1-Ab) raised in goat was obtained from Santa Cruz Biotechnology Inc. (Heidelberg, Germany). The secondary antibodies, CY3-labeled goat anti-rabbit IgG (GARCY3) and Alexa Fluor 488-labeled chicken anti-goat IgG (CAG-488F, A21467), were obtained from Jackson ImmunoResearch Laboratories Inc. (West Grove, PA) and Life Technologies (Darmstadt, Germany), respectively.

### 2.7. Immunofluorescence

Fixation of the tissue with 4% paraformaldehyde, cutting of tissue cryosections, and immunostaining protocol were described in detail earlier [61]. In brief, 4-*μ*m thick cryosections of fixed intestinal tissue were cut and collected on microscope slides. Following rehydration, antigen retrieval was performed by heating the sections in 10 mM citrate buffer (pH 6.0) in a microwave oven and incubating them in Triton-X-100-containing PBS solutions. The sections were blocked by incubation with 1% bovine serum albumin (in PBS) and incubated overnight in a refrigerator with PBS containing affinity-purified rSGLT1-Ab (1 : 500). Then, the sections were rinsed and incubated for 60 min at room temperature with the secondary antibody GARCY3. For immunostaining with GLP-1-Ab, sections were similarly processed with GLP-1-Ab and the secondary antibody CAG-48. After staining, the sections were overlaid with Vectashied (Vector Laboratories Inc., Burlingame, CA, USA), covered with a coverslip, and sealed with nail polish. Stained slides were photographed with a Keyence Biorevo BZ-9000 Microscope (Keyence Corporation, Osaka, Japan). The full focus function of the BZ-II Image Analysis Application was used to merge captured images into a single image.

### 2.8. Semiquantification of rSGLT1-Ab Staining of Brush-Border Membranes (BBM)

For comparison of staining intensities of rSGLT1-Ab immunoreaction ileum from sham-operated rats and interposed ileum were proceeded on identical slides. Exposure time and rSGLT1-Ab concentration were adjusted to a range in which the observed fluorescence staining intensity was proportional to the concentration of the primary antibody. Semiquantification was performed with program ImageJ 1.46r supplied by the National Institute of Health (Bethesda, Maryland, USA). To compare staining of BBM in different samples, fluorescence images with 100-fold magnification as shown in Figures 7(d) and 7(e) were used. Staining of the BBM was quantified by analyzing the staining intensity of bands with constant width covering BBM segments. The obtained staining intensities were normalized to the length of the analyzed bands which was determined in parallel. The total length of BBM in a given cross section was determined using the ImageJ function. The heights of villi were measured in longitudinally sectioned villi.

### 2.9. Electron Microscopy

Ileal segments were fixed in 4.5% glutaraldehyde in 0.1 M phosphate buffer, pH 7.2. After washing with this phosphate buffer, specimens were fixed for 1 h with 1% osmium tetroxide in phosphate buffer and washed with water. Specimens were dehydrated in ascending concentrations of ethanol including en-bloc contrasting using 2% uranyl acetate in 70% ethanol for 1 h, embedded in Epon812, and ultrathin sections were prepared. Sections were poststained with 2% uranyl acetate and 0.2% lead citrate and observed using a LEO AB 912 transmission electron microscope (Zeiss NTS, Oberkochen, Germany).

### 2.10. Measurements of Blood Glucose

Blood glucose was measured between 9 and 10 a.m. in nonstarved rats or in rats after an 18-hour fast. Blood (2 *μ*L) was collected from the tail vein and analyzed using the amperometric glucose oxidase method (glucose meter, ACCU-CHEK Aviva).

### 2.11. Measurement of Plasma Insulin

For monitoring insulin levels after overnight starvation and 5 or 15 min after application of d-glucose (3 g d-glucose/kg body weight) by oral gavage, blood was collected from the portal vein. Insulin measurements in the plasma were performed with the insulin ELISA number 80-INSMSU-E01 from ALPCO Diagnostics (Salem NH, USA) according to the manufacturer's instructions.

### 2.12. Oral Glucose Tolerance Test (OGTT)

After 18 h of fasting, blood glucose was measured at 10 a.m. (time 0). Then 3 g d-glucose/kg body weight was applied by oral gavage and blood glucose was determined 15, 30, 60, 90, 120, and 180 min after administration.

### 2.13. Insulin Tolerance Test (ITT)

At 10 a.m., 0.75 IU/kg human insulin (Insuman rapid, Sanofi-Aventis Deutschland, Frankfurt, Germany) was administered intraperitoneal to rats after an 18-hour fast. Blood glucose was measured before insulin injection and 30, 60, and 90 min after insulin injection.

### 2.14. GLP-1 ELISA

To determine GLP-1 in plasma, blood was obtained from tails before and 15 min after the rats had been challenged with 3 g d-glucose/kg body weight by oral gavage. Total GLP-1 was measured using the GLP-1 total ELISA EZGLP1T-36k kit from EMD Millipore, Merck KGaA (Darmstadt, Germany).

### 2.15. Uptake of *α*-Methyl-d-glucopyranoside by Everted Small Intestinal Rings

Uptake measurements were performed as described [11]. In brief, rats were starved for 18 h and sacrificed between 10 a.m. and noon. The small intestines were removed and perfused with Krebs-Ringer buffer (25 mM HEPES, 108 mM NaCl, 4.8 mM KCl, 1.2 mM KH_2_PO_4_, 1.2 mM CaCl_2_, pH 7.4, and 37°C). The small intestine was everted and the regions selected for uptake measurements (Figure 1(a)) were cut into four segments of 1 cm length. The segments were incubated for 2 min at 37°C with Krebs-Ringer buffer containing 10 *μ*M of the SGLT1 specific glucose analogue [^14^C]*α*-Methyl-d-glucopyranoside (AMG), with or without 0.2 mM of the SGLT1-specific inhibitor phlorizin. Uptake was stopped by transferring the segments into ice cold Krebs-Ringer buffer containing 0.2 mM phlorizin. After washing with ice cold Krebs-Ringer buffer, the length of the individual segments was measured under a microscope, and the segments were solubilized with Soluene-350 (Perkin Elmer Inc., Waltham, MA, USA). Radioactivity was analyzed by liquid scintillation counting.

### 2.16. Statistical Analysis

**Figure 2:**
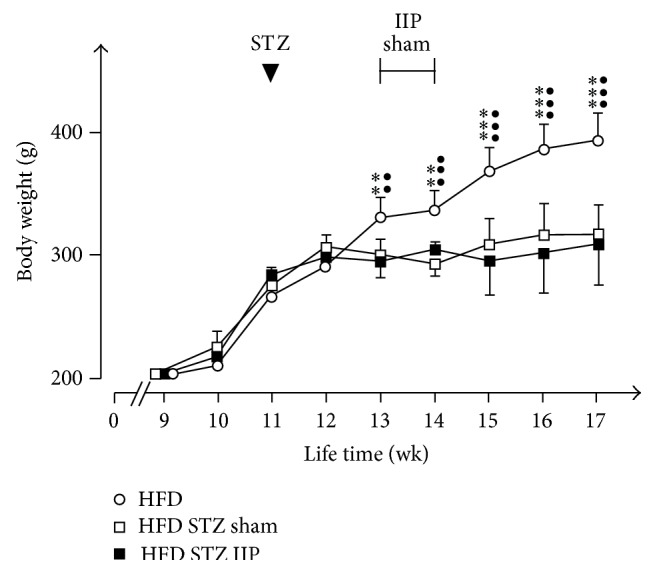
Effects of HFD, STZ treatment, and IIP surgery on body weight. All 27 animals received HFD starting with their 9th week of life and were treated with STZ after the 11th week of life. Sham or IIP surgery was performed in groups of 9 animals. Mean values ± SEM are indicated. ^●●^ *p* < 0.01 and ^●●●^ *p* < 0.001 difference between HFD and HFD STZ sham animals; ^*∗∗*^ *p* < 0.01 and ^*∗∗∗*^ *p* < 0.001 difference between HFD and HFD STZ IIP animals.

**Figure 3:**
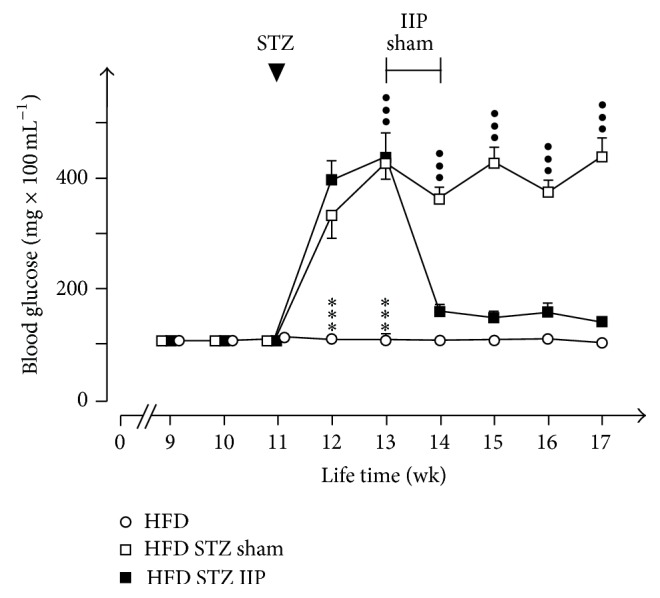
Effects of HFD, STZ treatment, and IIP surgery on blood glucose levels in nonfasting rats. Blood glucose was measured between 9 and 10 a.m. in rats that had free access to HFD and water. Mean values ± SEM of 9 animals are indicated. ^●●●^ *p* < 0.001 difference between HFD and HFD STZ sham animals and ^*∗∗∗*^ *p* < 0.001 difference between HFD and HFD STZ IIP animals.

**Figure 4:**
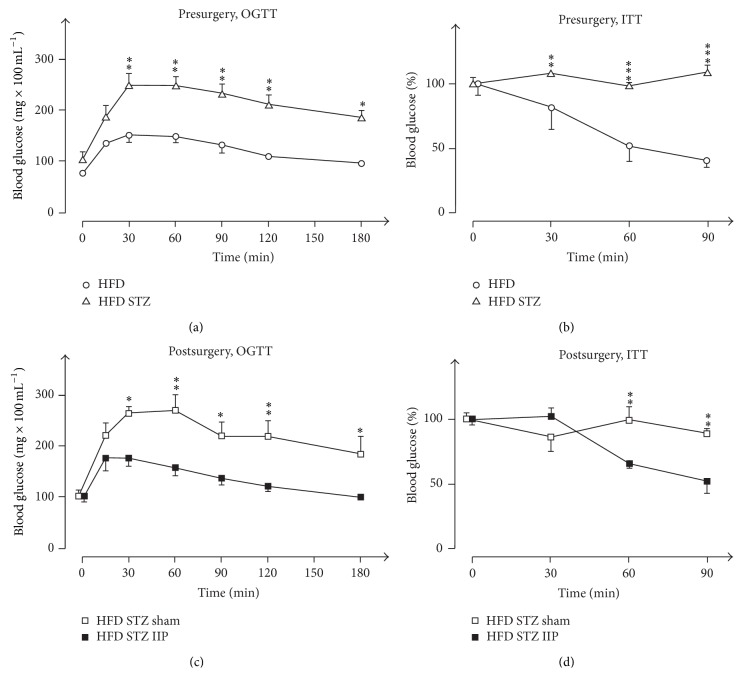
Effects of STZ treatment ((a), (b)) and IIP surgery ((c), (d)) on oral glucose tolerance ((a), (c)) and insulin tolerance ((b), (d)). Before surgery: oral glucose tolerance tests (OGTTs) in (a) and insulin tolerance tests (ITTs) in (b) were performed during the 12th week of life in 9 animals that did not receive STZ (HFD) and in 18 animals that were treated with STZ (HFD STZ). After surgery: OGTTs (c) and ITTs (d) were performed during the 15th week of life in STZ-treated animals that had been sham-operated (HFD STZ sham, 9 animals) and in STZ-treated animals that had been IIP-operated (HFD STZ IIP, 9 animals). Mean values ± SEM are indicated. ^*∗*^ *p* < 0.05, ^*∗∗*^ *p* < 0.01, and ^*∗∗∗*^ *p* < 0.001.

**Figure 5:**
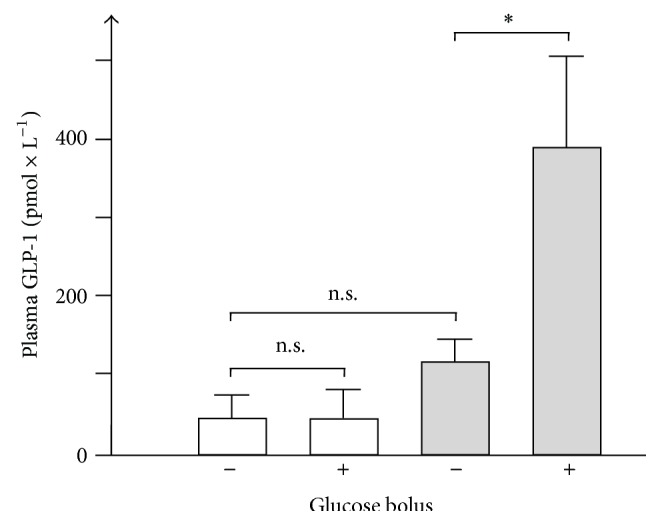
Effect of IIP on GLP-1 secretion after glucose gavage. In rats on HFD with STZ-induced diabetes either sham operation (white columns) or IIP surgery (grey columns) was performed during their 13th week of life. Four weeks later, the mice were force-fed with PBS (glucose bolus −) or with PBS containing glucose (glucose bolus +). Fifteen minutes later, total GLP-1 was measured in venous blood plasma obtained from the tail. Mean values ± SEM from six animals are presented. ^*∗*^ *p* < 0.05.

**Figure 6:**
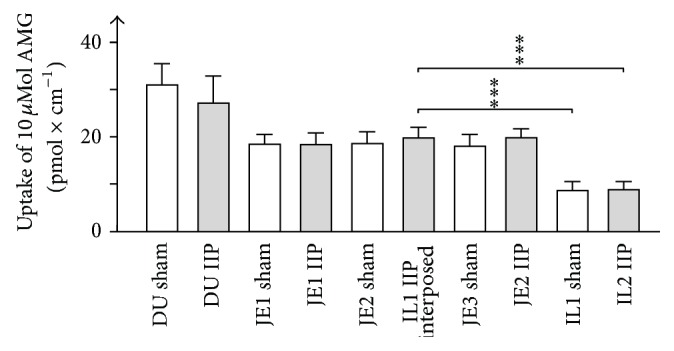
SGLT1-mediated glucose uptake in small intestinal segments of rats on HFD with STZ-induced diabetes after sham operation (open columns) or IIP surgery (closed columns). Sham operation or IIP was performed in the 13th week of life and the* ex vivo* uptake measurements were performed in the 18th week of life. Uptake of 10 *μ*M [^14^C]AMG inhibited by 200 *μ*M phlorizin was measured in everted small intestinal segments obtained from the regions indicated in Figure 1(a). Mean values ± SEM from 6 animals are indicated. ^*∗∗∗*^ *p* < 0.001.

GraphPad Prism 5.0 software (GraphPad Software Inc., La Jolla, USA) was used for statistical analyses. Mean values ± SEM are indicated. For changes in body weight or plasma glucose concentrations over time (Figures 2, 3, and 4) significances of differences were analyzed by 2-way ANOVA followed by Bonferroni's post hoc comparisons. For comparison of GLP-1 concentrations and glucose uptake in more than two samples (Figures 5 and 6), the 1-way ANOVA test with Tukey's post hoc comparison was performed. For comparison of two mean values unpaired Student's* t*-test was used. *p* < 0.05 was considered statistically significant.

## 3. Results

### 3.1. Effects of STZ Treatment, IIP, and Sham Surgery on Body Weight of Rats on HFD

The experiments were performed with male Lewis rats (*n* = 27) fed with HFD between the 9th and the 18th weeks of life (Figure 1(b)). After two weeks on HFD, the starting mean body weight of 204 ± 9 g had increased to 276 ± 2.5 g (*p* < 0.001) (Figure 2). Eighteen of the 27 animals were treated with STZ in their 11th week of life. They underwent IIP or sham surgery in their 13th week of life. Four weeks after surgery both groups showed a similar body weight that was slightly increased compared to two weeks after HFD (313 ± 6.7 g) (Figure 2). The body weight gain of these animals was significantly smaller compared to untreated control animals on HFD (395 ± 6.6 g, *p* < 0.001). A similar reduction of weight increase during the first weeks after IIP and sham surgery has been described for rats on standard diet with low-dose STZ-induced diabetes [41] and for a genetic type 2 diabetes mellitus rat model [48]. However, in these experiments, a reduction of body weight after IIP surgery compared to sham surgery was clearly evident after longer postsurgery periods [12, 26, 29, 30, 37, 38, 40, 41, 43, 44, 46, 47, 58, 62].

### 3.2. Effects of STZ Treatment and IIP Surgery on Blood Glucose in Rats on HFD

Consistent with the previous results [11], we observed that HFD for eight weeks did not increase nonfasting blood glucose (Figure 3). In 11-week-old rats on HFD, blood glucose was 116 ± 3 mg/dL (*n* = 27). Two weeks after STZ treatment, nonfasting blood glucose was significantly increased to 434 ± 24 mg/dL (*n* = 18) (Figure 3). Sham operation performed three weeks after STZ treatment did not affect high blood glucose levels during the following three weeks (439 ± 34.8 mg/dL, *n* = 9). In contrast, IIP surgery significantly lowered nonfasting blood glucose to 142 ± 8.7 mg/dL (*n* = 9) within three weeks after surgery (Figure 3).

### 3.3. Effects of STZ Treatment on Glucose Tolerance, Plasma Insulin, and Insulin Sensitivity

OGTT and ITT confirmed the previously described observations that the employed protocol for STZ treatment of rats on HFD impaired glucose tolerance and decreased insulin sensitivity [11]. Presurgery OGTT measurements of plasma insulin and ITT were performed on rats in the 12th week of life with and without diabetes (Figure 1(b)). In nondiabetic rats, glucose values during OGTT increased significantly to 151 ± 13.1 mg/dL after 30 min and decreased to the starting levels (95.7 ± 1.5 mg/dL) after 3 h (Figure 4(a)). In STZ-treated diabetic rats, blood glucose increased twice during OGTT compared to nontreated animals. 3 h after glucose gavage, blood glucose was still significantly higher (186 ± 14 mg/dL versus 95.7 ± 1.5 mg/dL, *p* < 0.001).

Plasma insulin levels in blood from the portal vein were measured after overnight starvation and 5 min and 15 min after oral gavage with d-glucose. After overnight starvation, the plasma insulin in nondiabetic rats was higher compared to the STZ-treated animals (0.44 ± 0.07 ng/mL versus 0.09 ± 0.01 ng/mL, *p* < 0.01, *n* = 4 each). 5 min after application of d-glucose, insulin levels were increased in nondiabetic rats (1.81 ± 0.10 ng/mL, *n* = 4, *p* < 0.001) and in STZ-treated rats (0.33 ± 0.01 ng/mL, *n* = 4, *p* < 0.001). 15 min after glucose application, similar insulin concentrations were observed in nondiabetic and STZ-treated diabetic rats (0.44 ± 0.05 ng/mL versus 0.47 ± 0.04 ng/mL). The data indicate that the insulin secretion by *β* cells was strongly reduced after STZ treatment but not abolished completely.

After intraperitoneal injection of 0.75 IU/kg insulin in nontreated rats during ITT, blood glucose decreased within 90 min from 98.5 ± 1.8 mg/dL (100%) to 41.5 ± 3.9 mg/dL (42%) (Figure 4(b)). In the STZ-treated rats, the same amount of insulin did not provoke a decrease in blood glucose indicating that these rats were insulin-resistant (Figure 4(b)).

### 3.4. Effects of IIP Surgery on Glucose Tolerance and Insulin Sensitivity

Diabetic rats received IIP or sham surgery in the 13th week of life (Figure 1(b)). OGTT and ITT after surgery were performed in the 15th week of life (four weeks after STZ treatment). In sham-operated animals, similar results in OGTT and ITT were obtained (Figures 4(c) and 4(d)) as in nonoperated animals in the 12th week of life (one week after the STZ treatment) (Figures 4(a) and 4(b)). This indicates that STZ-induced glucose intolerance and insulin insensitivity were not changed by sham surgery or time. In the 15th week of life and two weeks after IIP surgery, glucose excursion during OGTT was comparable to nondiabetic animals on HFD (compare Figure 4(c) with Figure 4(a)). Remarkably, blood glucose measured 15 min after glucose gavage which mainly represents intestinal glucose absorption was not decreased significantly (Figure 4(c)). This is in contrast to DJB (see Figure 4(c) in [11]) and suggests that intestinal glucose absorption is not decreased after IIP. After IIP, insulin sensitivity was improved. During ITT, the injection of 0.75 IU/kg insulin decreased blood glucose after 90 min from 99.7 ± 2.5 mg/dL to 49.5 ± 2.2 mg/dL (Figure 4(d)). The data show that IIP surgery improved glucose tolerance and insulin sensitivity in rats with T2LD. Improved glucose tolerance and insulin sensitivity following IIP surgery have been previously reported in two nonobese genetic diabetic models: the Goto-Kakizaki-rats [13, 26, 38, 39] and the Zucker rats [47].

### 3.5. Effect of IIP Surgery on Glucose-Induced GLP-1 Secretion in Diabetic Rats

To verify that IIP surgery increased glucose-stimulated GLP-1 secretion in the employed model of type 2 like diabetes as reported by Strader and coworkers [37, 38, 40, 42], we measured GLP-1 concentrations in systemic blood before and 15 min after glucose gavage. Sham-operated and IIP-operated animals were compared in the 17th week of life four weeks after surgery (Figure 5). In sham-operated animals, no increase in plasma GLP-1 levels 15 min after glucose gavage was observed. After IIP surgery compared to sham surgery, an elevated basal GLP-1 level was measured; however, this difference was not significant (*p* = 0.057). 15 min after glucose gavage of animals with IIP surgery, the GLP-1 concentration in the blood was about 3-fold increased. This concentration was 9-fold higher compared to sham-operated animals. The data are consistent with previous reports which indicate that IIP surgery increases the glucose-dependent secretion of GLP-1 in small intestine of diabetic rats [26, 38, 39, 42, 44].

### 3.6. Glucose Absorption in Small Intestine before and after IIP Surgery

Glucose absorption in small intestine is mediated by glucose uptake into enterocytes across the brush-border membrane (BBM) via the Na^+^-d-glucose cotransporter SGLT1 followed by glucose efflux across the basolateral membrane of the enterocytes which is mediated by the passive glucose transporter GLUT2 [24]. SGLT1-mediated glucose uptake via the brush-border membrane is rate limiting for small intestinal glucose absorption [25]. The highest expression of SGLT1 is observed in duodenum, followed by jejunum and ileum [11]. Previously we reported that glucose absorption in small intestine after DJB surgery was reduced not only due to the shortened small intestinal alimentary path but also due to a downregulation of SGLT1 in the remaining jejunal part [11]. To determine whether small intestinal glucose absorption is also reduced after IIP surgery, we determined SGLT1-mediated transport activity in the small intestinal regions of sham- and IIP-operated rats in the 18th week of life in the small regions indicated in Figure 1(a). We measured phlorizin-inhibited uptake of 10 *μ*M [^14^C]AMG by everted small intestinal fragments (Figure 6). This transport activity is almost exclusively mediated by SGLT1 [24, 25]. In sham-operated rats, the highest phlorizin-inhibited AMG uptake related to intestinal length was observed in duodenum. AMG uptake in the three analyzed jejunal regions of sham-operated rats was similar and was about 30% smaller compared to duodenum. AMG in ileum of sham-operated animals was about 70% smaller compared to duodenum. After IIP surgery, AMG transport measured in duodenum, in jejunal part proximal to the interposed ileal fragment (JE1 IIP in Figure 6), and in jejunal part distal to the interposed ileal fragment (JE2 IIP in Figure 6) was not influenced by IIP surgery. AMG transport in the ileal region distal to the dissected ileum (IL2 IIP in Figure 6) was similar to transport in the ileal regions analyzed in sham-operated animals. Importantly, phlorizin-inhibited AMG uptake per intestinal length of the interposed ileal fragment (see Figure 6, IL1 IIP interposed) was similar to uptake measured in jejunum. Thus, AMG uptake per intestinal length was increased 2.3-fold after translocation of ileum. The data indicate that IIP surgery did not decrease glucose absorption in small intestine at variance with DJB [11].

### 3.7. Structural Changes of Epithelial Cells in the Interposed Ileum

**Figure 7:**
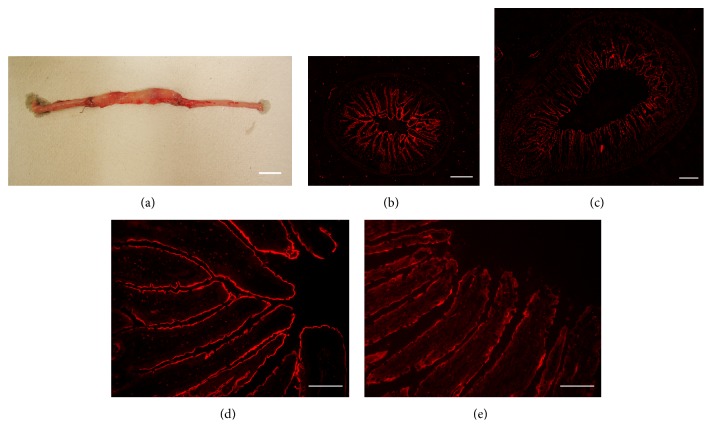
Macroscopy appearance of interposed ileum (a) and immunohistochemistry of the interposed ileum ((c), (e)) in comparison with the respective ileal segment of a sham-operated animal ((b), (d)). The samples were taken after the animals had been killed in the 3rd week after sham or IIP surgery. Immunostaining in (b)–(e) was performed with SGLT1-Ab. Representative images are shown. Bars: (a) 2 cm, (b) and (c) 500 *μ*m, and (d) and (e) 50 *μ*m.

**Figure 8:**
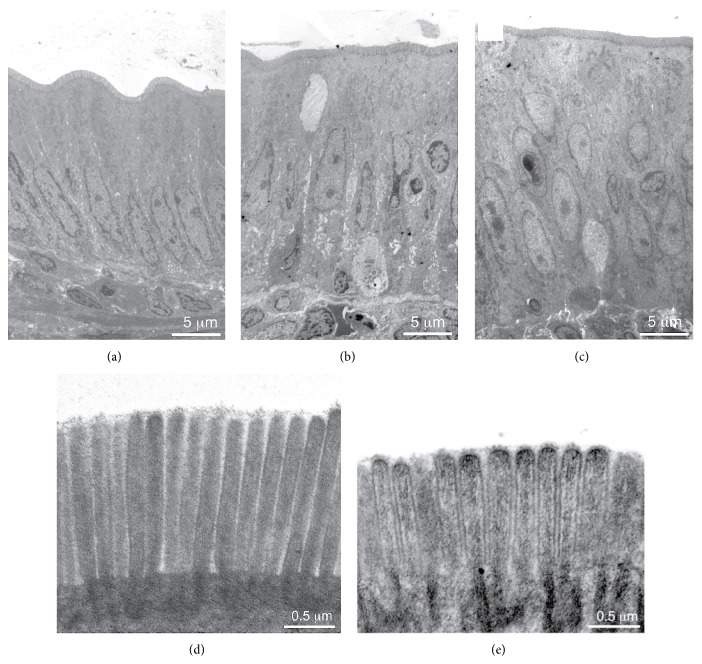
Electron microscopic appearance of the ileal epithelium of a sham-operated rat ((a), (d)) in comparison with the ileal epithelium of the interposed ileum ((b), (c), and (e)). The samples were taken in the 3rd week after sham or IIP surgery. (b) and (c) show epithelial layers of the interposed ileum with different degrees of hyperplasia; (d) shows microvilli of an epithelial ileal cell from a sham-operated animal; (e) shows microvilli of the luminal cell layer of interposed ileum.

**Figure 9:**
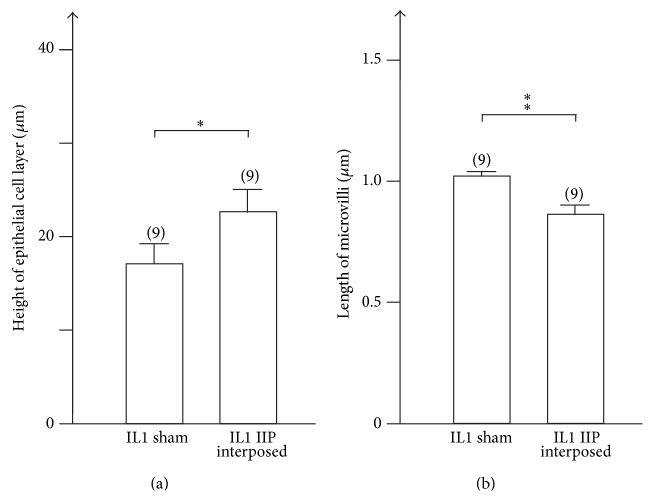
Effect of ileal interposition on height of the epithelial cell layer (a) and length of microvilli (b). Electron microscopic images from ileal epithelial cells of sham-operated animals and of interposed ileum similar to those shown in Figures 8(d) and 8(e) were employed to measure length of microvilli. The measurements were performed on 3 animals using three sections from different regions of each analyzed segment. For measurement of epithelial height (a) 11–13 individual measurements were performed on each section. Length of microvilli in a section was determined in 3 cells measuring 5 microvilli per cell. Mean values ± SEM. The number of analyzed sections is indicated in parenthesis. ^*∗*^ *p* < 0.05, ^*∗∗*^ *p* < 0.01.

Morphological changes of the interposed ileal segment after IIP surgery have already been described three decades ago and were also observed since [12, 58, 62, 63]. The interposed ileal segment has been reported to increase in weight and diameter [12, 58]. It has been described that the wet weight, the protein content, and the DNA content of the mucosa of the interposed ileal segment increase about 3-fold [63]. We made similar observations. Five weeks after IIP surgery, the diameter of the interposed ileal segment had increased 1.5 ± 0.1 times (12 determinations performed in 9 sections of 3 animals, *p* < 0.01 for difference). The length of the villi had increased 1.6 ± 0.1 times (30 determinations performed in 9 sections of 3 animals, *p* < 0.01 for difference), whereas the width of the villi did not change significantly (Figures 7(b)–7(e)). The length of the BBM per cross section had increased 4.8 ± 0.6 times (7 determinations performed in 3 animals, *p* < 0.001 for difference). We compared the epithelial cell layer of the interposed ileum with the respective sham-operated ileal segment using transmission electron microscopy. In sham-operated animals, the ileal surface was continuously covered by the typical monolayer of cylindrical enterocytes (Figure 8(a)). In contrast, only part of the interposed ileum was covered by a monolayer of enterocytes, whereas about 75% was covered by an epithelial layer containing 2 or more layers of nuclei indicating hyperplasia (Figures 8(b) and 8(c)). In sham-operated animals, we never observed more than one nucleus per “epithelium height” (56 determinations performed in 9 sections of 3 animals) but determined 1.8 ± 0.6 nuclei per “epithelium height” (56 determinations performed in 9 sections of 3 animals, *p* < 0.01 for difference) in regions of interposed ileum with hyperplasia. The mean height of enterocyte monolayer in sham-operated animals was 17.1 ± 2.2 *μ*m. This is significantly smaller compared with the mean height in interposed ileum with hyperplasia (22.7 ± 2.4 *μ*m) (Figure 9(a)). The microvilli of enterocytes in ileum of sham-operated animals appeared to be longer (Figure 8(d)) compared to microvilli of enterocytes in regions of interposed ileum with hyperplasia (Figure 8(e)). Significantly different values of 1.02 ± 0.04 *μ*m and 0.86 ± 0.04 *μ*m were determined (Figure 9(b)). The data indicate hyperplasia and a lower degree of cell surface differentiation of epithelial cells in the interposed ileum.

### 3.8. SGLT1 Protein at the Luminal Membrane of the Interposed Ileum

Using a specific antibody against rat SGLT1 (rSGLT1-Ab), we employed immunohistochemistry to compare the amounts of SGLT1 at the brush-border membrane (BBM) of the interposed ileum with the respective segment of sham-operated animals. With this antibody, we had previously obtained staining of the BBM of rat small intestine that could be blocked with antigenic peptide [61]. In Figures 7(b)–7(e) we compared rSGLT1-Ab staining of ileum from sham-operated animals with that of interposed ileum in IIP animals. The BBM of ileum from sham-operated animals was stained more strongly than BBM of the interposed ileum (Figures 7(b)–7(e)). Quantification of BBM immunostaining related to BBM length revealed 2.5 ± 1.5-fold higher staining intensity in ileum of sham-operated animals than in interposed ileum (80 measurements in 9 sections of 3 animals each, *p* < 0.01 for difference). Because the length of the BBM per cross section in interposed ileum was increased 4.8-fold compared to ileum of sham-operated animals (6 determinations in three animals each), 1.9-fold more SGLT1 protein was associated with the total BBM-surface of the interposed ileum compared to ileum of sham-operated rats. The similar 2.3-fold increase in SGLT1-mediated glucose uptake per intestinal length suggests that SGLT1 in the BBM of interposed ileum is functionally active.

### 3.9. GLP-1 Producing L-Cells in the Mucosa of Interposed Ileum

The increased glucose-induced release of GLP-1 from L-cells observed in animals after IIP surgery may be explained by an increased stimulation of L-cells due to contact with higher intraluminal glucose concentrations after surgery. To determine whether the number of L-cells in the mucosa in the interposed ileum was altered, we stained interposed ileum and the corresponding ileal fragment of sham-operated animals with antibody against GLP-1 and counted the immunoreactive L-cells per ileal cross sections. In ileum of sham-operated animals 9.2 ± 2.5 and in the interposed ileum 17.5 ± 1.2 L-cells per ileal cross section were counted (15 determinations in 3 animals each, *p* < 0.01 for difference). The data are consistent with previous reports [37, 39, 64]. They suggest that an increase in the number of L-cells in the interposed ileum contributes to the higher glucose stimulation of GLP-1 secretion.

## 4. Discussion

In the present study, we show in rats with an experimental induced type 2 like diabetes (T2LD) that weight-independent improvement of diabetic control observed after IIP cannot be explained by changed glucose absorption. After glucose uptake, a slowed down increase of blood glucose was expected because interposition of the distal ileum with low expression of SGLT1, which has been shown to be rate limiting for small intestinal glucose absorption [25], was supposed to decrease the rate of glucose absorption. However, we show that SGLT1-mediated glucose uptake in the interposed ileal segment is upregulated to a similar level as in the jejunum. The upregulation of glucose uptake is due to an increase of total SGLT1 protein at the enlarged luminal surface of the interposed ileal segment.

To determine the direct role of SGLT1-mediated glucose absorption for weight-independent improvement of glycemic control by bariatric surgery, we investigated the effects of IIP surgery in rats with an experimental type 2 like diabetes. We chose IIP because, unlike other bariatric procedures, it does not alter regulatory mechanisms governed by the stomach [14, 15]. In addition, IIP does not alter the length of the alimentary pass in contrast to the DJB and RYGB. In rats on HFD, diabetes was induced by a single injection of STZ. Although the employed model of experimental diabetes does not depict diabetes mellitus type 2 perfectly, close similarities to the type 2 diabetes were observed. Fasting blood glucose was not increased significantly; however, an increased and broadened peak of blood glucose was observed in the OGTT. In addition, the insulin sensitivity was decreased in the ITT. Furthermore, a reduced but not completely abolished function of pancreatic *β* cells was demonstrated. Fasting plasma insulin in the portal vein and the increase of insulin after oral gavage with glucose were reduced but not abolished. Two weeks after IIP, the pathologic glucose tolerance had improved and the insulin sensitivity increased resembling the parameters of nondiabetic animals. Four weeks after IIP, GLP-1 secretion after glucose gavage had increased. Because in our model the improvement in glycemic control was observed at a time when the body weights of the sham-operated and IIP-operated animals were similar, overall metabolic changes are not supposed to cause the observed improvement of diabetes. Contributions of changes in feeding behavior and energy expenditure cannot be excluded because these parameters were not analyzed.

To determine effects of IIP on glucose absorption, we compared phlorizin-inhibited uptake of AMG in various parts of small intestine five weeks after sham operation and IIP surgery. Phlorizin-inhibitable AMG uptake is mediated by the Na^+^-d-glucose cotransporter SGLT1 in the BBM of the enterocytes. It is proportional to the rate of small intestinal glucose absorption because transport via SGLT1 across the BBM is rate limiting for glucose absorption [25]. We performed the uptake measurements in various parts of small intestine including the interposed ileal segment in IIP-operated animals. The measured uptake was related to the length of the analyzed small intestinal segments because, in contrast to intestinal mass, intestinal protein, or intestinal DNA, the length of the ileum remains almost constant after IIP. Because the SGLT1-mediated uptake of AMG in the interposed ileal segment per unit length was increased to a value similar to uptake in jejunum of sham-operated animals, the rate of small intestinal glucose absorption remained largely unchanged after IIP.

Consistent with previous reports, we observed an increase in the secretion of GLP-1 in response to glucose gavage after IIP [26, 37–40, 42, 44–48]. The effect of IIP on GLP-1 secretion by L-cells is probably critically involved in the body weight-independent therapeutic effect of IIP surgery on diabetes. This supposition is supported by data showing that the improvement in the OGTT after IIP was blunted in the presence of an antagonist of the GLP-1 receptor [43]. IIP induces body weight reduction due to the anorexigenic effect of GLP-1. The weight reduction may contribute to long-term improvement of diabetes. Increased secretion of the anorexigenic enterohormone PYY after IIP [30, 37, 46] may also be involved in weight reduction and long-term improvement of diabetes. Because an increase in glucose-dependent GLP-1 secretion has also been observed after other bariatric procedures, such as DJB and RYGB [26, 37, 38], an increase of GLP-1 secretion is probably also involved in the weight-independent antidiabetic effects of these procedures. However, one or more additional antidiabetic mechanisms may contribute, at least in the case of RYGB surgery. A recent study reports improvement in glycemic control after RYGB surgery in mouse models with functional GLP-1 receptor deficiency [31].

The biological and biomedical functions of GLP-1 strongly support its playing a pivotal role in the weight-independent improvement of diabetes after IIP. GLP-1 has been shown to be a highly effective antidiabetic enterohormone and GLP-1 analogues are used for antidiabetic therapy [65]. GLP-1 increases glucose-dependent insulin secretion, exhibits trophic effects on *β* cells, inhibits glucagon secretion, inhibits glucose production in liver, increases glucose uptake in heart, inhibits gastric emptying and postprandial gastrointestinal motility, and reduces appetite [21, 22, 66, 67]. A unifying hypothesis to explain why different bariatric procedures lead to an increase in glucose-induced GLP-1 secretion is that all procedures lead to an increased glucose activation of L-cells after glucose-rich meals. After IIP, the terminal ileum containing most L-cells is located within the proximal jejunum where the intraluminal glucose concentration is high. In DJB and RYGB, duodenum and the proximal part of jejunum are excluded from the alimentary path so that the amount of glucose reaching the ileum after a glucose-rich meal is increased. This effect is enhanced after DJB and RYGB by downregulation of SGLT1 in the jejunal segment that remains in the alimentary path [11, 32, 68]. Shortening of the food retention time in the stomach and changes in small intestinal motility after RYGB are also thought to increase the glucose concentration in the ileum after glucose-rich meals.

Bariatric procedures may change intestinal morphology and the subcellular organization of enterocytes as well as the expression of functional proteins in enterocytes. These changes may occur in response to surgery-induced impairment of innervation and/or vascular supply as well as altered nutrient signals that regulate expression of transporters and of metabolic enzymes in the enterocytes. Morphometric measurements after RYGB surgery indicated an increase in bowel width, villus height, and crypt depth in the common alimentary limb [32, 67]. Proliferation of enterocytes in the crypts was observed [32]. Several morphological changes in the interposed ileum after IIP have been described: an increase in weight and diameter [12, 58], broadening of the mucosal and muscular layers [37], and increase in villus height [37]. The observed increase in a marker for epithelial proliferation indicates hyperplasia of the enterocytes [12, 37, 63]. In the present study, we confirm the increase in diameter, the thickening of total bowel wall, and the increase in villus height. With electron microscopy, we observed that about 70% of the intestinal mucosa showed thickening of the epithelial layer and that the monolayered cylindrical epithelium was partially transformed into a two- or triple-row epithelium. We further observed that the microvilli of enterocytes of the interposed ileum were shorter compared to the sham-operated ileum. Semiquantitative analysis of SGLT1-Ab immunoreactivity revealed that the amount of SGLT1 protein per unit length of the BBM decreased 2.5-fold after interposition. However, because the surface of the BBM lining the lumen of the ileum had increased 4.8-fold, the amount of SGLT1 lining the luminal surface showed a 1.9-fold increase. This value is similar to the observed 2.3-fold increase of SGLT1-mediated uptake per small intestinal length, suggesting similar plasma membrane incorporation and activity of SGLT1 in both cases. Noteworthy is the increased number of GLP-1-secreting L-cells per cross section after interposition of the ileum. This should contribute to the increased GLP-1 secretion after glucose-rich meals.

## 5. Conclusions

After IIP surgery, morphology and function of the interposed ileal segment change. For example, diameter and luminal surface containing SGLT1 increase, and glucose absorption capacity of the interposed ileal segment becomes similar to jejunum. Thus, improvement of weight-independent glycemic control following IIP surgery is independent of a change in glucose absorption. The observed increase of glucose-induced GLP-1 secretion after IIP due to increased number of L-cells in the interposed ileal segment and their enhanced exposition to nutrients including glucose provides a plausible explanation for the weight-independent antidiabetic effect of IIP. Whereas bypass procedures that are combined with partial or total removal of the stomach like RYGB may be best suited to treat morbid obesity without and with diabetes, a surgical procedure that does not change the stomach, such as DJB or IIP, may be best suited to treat type 2 diabetes of nonobese or slightly overweight patients.
